# Dietary habits and nutritional status of medical school students: the case of three state universities in Cameroon

**DOI:** 10.11604/pamj.2020.35.15.18818

**Published:** 2020-01-23

**Authors:** Fala Bede, Samuel Nambile Cumber, Claude Ngwayu Nkfusai, Mbinkar Adeline Venyuy, Yunga Patience Ijang, Emerson Njokah Wepngong, Agatha Tanya Nguti Kien

**Affiliations:** 1 Faculty of Medicine and Biomedical Sciences, University of Yaoundé I, Yaoundé, Cameroon; 2 Cameroon Baptist Convention Health Services (CBCHS), Yaoundé, Cameroon; 3 Institute of Medicine, Department of Public Health and Community Medicine (EPSO), University of Gothenburg, Gothenburg, Sweden; 4 Faculty of Health Sciences, University of the Free State, Bloemfontein, South Africa; 5 School of Health Systems and Public Health, Faculty of Health Sciences, University of Pretoria, Pretoria, South Africa; 6 Department of Microbiology and Parasitology, Faculty of Science, University of Buea, Buea, Cameroon; 7 Collaboration for Research Excellence in Africa (CORE Africa); 8 Department of Public Health, School of Health Sciences, Catholic University of Central Africa, Yaoundé, Cameroon; 9 College of Technology, Department of Food Sciences and Nutrition, University of Bamenda, Bamenda, Cameroon

**Keywords:** University students, medical students, weight, height, nutritional status, body mass index, Cameroon

## Abstract

**Introduction:**

Malnutrition is a major risk factor of cardiovascular and metabolic diseases and therefore the importance of good dietary practices and balanced diet cannot be overemphasized. University students tend to have poor eating practices which is related to nutritional status. The objective of our study was to assess the dietary practices of medical students, determine the prevalence of malnutrition among medical students and factors associated with malnutrition.

**Methods:**

We carried out a cross-sectional study from December 2013 to March 2014 involving 203 consenting students in the Faculty of Medicine and Biomedical Sciences of the University of Yaoundé I, Faculties of Health Sciences of the Universities of Bamenda and Buea. A three-part questionnaire (socio-demographic profile, eating practices, and anthropometric parameters). Data was analysed using SPSS 18.0. Frequencies and percentages were determined for categorical variables. Means and standard deviations (mean ± SD) were calculated for continuous variables. Fischer’s exact test was used to compare the categorical variables. Statistical significance was set at p ≤ 0.05.

**Results:**

Males constituted 44.3% of respondents. The mean age was 20.8 ± 1.6yrs. Most students had a monthly allowance of less than 20 000frs (34 USD) and 59.1% lived alone. Most students (49.8%) reported taking two meals a day with breakfast being the most skipped meal while supper was the meal most consumed by students. Snacking was common among these students as 40.8% admitted consuming snacks daily. Daily intake of milk, fruits, vegetable and meat were low (6.2%, 4.3%, 20.0% and 21.3% respectively). The BMI status of students was associated with gender (p=0.026).

**Conclusion:**

Our findings showed a high prevalence of malnutrition of 29.4% based on BMI (underweight 4.9%, overweight 21.6% and obesity 3.0%) among second year medical students of these three state universities. Irregular meals, meal skipping, low fruit, vegetable and milk consumption, high candy, fried foods and alcohol intakes were found to be poor eating practices frequent among these students. Our findings therefore suggest the need for coordinated efforts to promote healthy eating habits among medical students in general and female medical students in particular (and by extension youths in general) as a means of curbing malnutrition among youths.

## Introduction

Nutritional status could be seen as the combination of an individual’s health as influenced by intake and utilization of nutrients and determined from information obtained by physical, biochemical and dietary studies [1]. Eating habits refers to why and how people eat, which foods they eat and with whom they eat, as well as the ways people obtain, store, use and discard food [2]. Poor nutritional status can be defined by the states of under-nutrition or over-nutrition. Many investigations have shown that among young people there is a high prevalence of malnutrition and risk factors of cardiovascular system diseases and metabolic diseases. The main reasons for these disturbances are inadequate food intake and poor dietary habits. Universities can be an ideal setting for preventive intervention programs particularly medical schools where future health personnel are trained. Medical students we believe are role models among their peers and stand a better chance of influencing and making them adopt good dietary habits. Studies have been carried out among university students [3-7] but none to the best of our knowledge on eating habits and nutritional status of medical school students in Cameroon. It was in this light that the study was carried out. We sought to: describe eating practices of medical students in Cameroon, determine the prevalence of malnutrition (undernutrition, overweight and obesity) among medical students and factors associated with malnutrition.

## Methods

The objectives of the study were to assess the dietary practices of medical students, determine the prevalence of malnutrition (undernutrition, overweight and obesity) among medical students and factors associated with malnutrition. The study was initially planned to include all the four state owned medical schools (University of Buea-UB, University of Bamenda-UBa, University of Yaoundé I-UYI and University of Douala-UDla) at the time, however due to inability to get an administrative authorization data was not collected for the medical school of UDla.

**Study setting:** the University of Bamenda was created on 14^th^ December 2010. The main campus is in Bambili, North-West Region of Cameroon. The University of Bamenda has two faculties and three professional schools of which the Faculty of Health Sciences is one. This faculty has several departments: Medical Laboratory Sciences, Nursing Science and Medicine. The University of Buea is located in Buea in South-West Region of Cameroon. It was founded as a university centre in 1985 and it became a full university in 1992. The Faculty of Health Sciences of the University of Buea was created by a presidential decree No. 93/034 of 19 January 1993. Since then, the two departments of Nursing and Medical Laboratory Science. In October 2006, the Minister of Higher Education authorized the start of the field of medicine. The Faculty of Medicine and Biomedical Sciences (FMBS) started as University Center for Health Sciences (popularly known by its french acronym CUSS - Centre Universitaire des Sciences de la Santé) in 1969 within the then Federal University of Cameroon, with 40 students and 6 permanent teachers. The first director was Pr. Gottlieb Lobe Monekosso. Today, the FMBS has grown not only to offer medicine as a program but also pharmacy, dentistry, medical specialisation programs and paramedical training with a student population of more than 1500.

**Ethical considerations:** ethical approval for the study was sought from the National Ethics Committee of Cameroon (Reference No: 247/14). This was a non-invasive study which entailed no risks or harm to the participants but required them to reveal some personal information. Consent was sought from participants following detailed explanation of the study with each person given an opportunity to have any doubts clarified. This was a voluntary process without any form of coercion. Participants were free to withdraw from the study and any point in time and have their information collected not used. Any student identified with severe malnutrition was counseled and referred to get appropriate care. Confidentiality, anonymity and privacy of all information were guaranteed at all the levels of this study. Administrative authorization was sought from deans of the different faculties before data collection was initiated.

**Study design and duration:** the study was a cross-sectional study. This study was conducted over a period of four months (from December 2013 to March 2014).

**Study population:** all students registered in the second year (2013/2014 academic year) of the medical program of all state universities offering a program in medicine in three public universities of the Republic of Cameroon (FMSB-Yaoundé, FHS-Buea and FHS-Bamenda). Second year medical students chosen because we believe compared to first year students they had fully immersed in university settings and adopted dietary lifestyles different from that of family homes. They were preferred to others because of reasons of availability during the period of the study.

**Inclusion and exclusion criteria:** the study included registered students of the 2013/2014 academic year in the second year of medical studies (medicine) in public universities (FMSB-Yaoundé, FHS-Buea and FHS-Bamenda) who gave consent. Students who were not in good health or had a history of any chronic medical condition were not included in the study.

**Sample size and sampling method:** the sample size (n) required for the study was calculated using the formula below (with correction for finite population):

$$n=\frac{m}{1+\frac{m}{N}}$$

where:

$$m=\left[p*(1-p)*Z_{\left(1-\frac{\alpha }{2}\right)}^{2}\right]/\varepsilon^{2}$$

and N was the total number of medical students in second year in the three state universities from where data was collected for our study. In this formula, N = 240 (FMBS=120, FHS-UB=70 and FHS-UBa=50), Ρ is the proportion of students with malnutrition (underweight, overweight or obese), ε the margin of error acceptable. We considered p = 0, 5 (50%) which makes it possible to obtain a maximum sample size, a precision ε = 0, 04 (4%) and α = 0, 05; the critical value resulting from the normal law was therefore:

$$Z_{\left(1-\frac{\alpha }{2}\right)}$$

= 1.96. Applying this formula, the required sample size therefore was 171. A total sample of 203 students (FMBS=107, FHS-UB=50 and FHS-UBa=46) obtained. They were selected by random sampling using the class list of each faculty as a sampling frame and taking into consideration the number of students per class in the different faculties (probability proportional to size).

**Study variables and operational definitions:** socio-demographic factors: for the purposes of this study, socio-demographic factors will include: age, gender, place of residence, religion, monthly allowance (income).

**Dietary habits (eating practices):** eating practices refer to meal patterns. Meal pattern refers to the frequency of major meals eaten in a day, where meals are eaten, snacking habits, meal skipping habits and daily food types and fluids consumption.

**Body mass index (BMI):** BMI referred to the relationship between current weight and current height (BMI = weight (kg)/Height (m^2^)). The BMI was classified according to the WHO international classification of BMI (Underweight: BMI < 18.5, Ideal: 18.5 ≤ BMI < 25.0, Overweight: 25.0 ≤ BMI < 30.0, Obese: BMI ≥ 30). Weight was measured using a calibrated electronic scale (CAMRY), with a range of 25 to 150Kg and an accuracy of 0.5Kg while a stadiometer was used to measure standing height of participants to the nearest centimeter.

**Waist circumference (WC):** WC refers to a measure of the abdominal circumference passing through the umbilicus as viewed from the front in nearest centimeter. The WHO classification for WC was used in analysis (Normal: WC<94 for male or WC<80 for females, Android Obesity: WC ≥ 94 for males or WC ≥ 80 for females). Waist circumference (WC) was measured with a flexible but inextensible measuring tape to nearest 0.1cm using the perimeter of the area through the umbilicus at end expiration.

**Waist-hip ratio (WHR):** refers to waist circumference (cm) divided by hip circumference (cm). The hip circumference (HC) was measured to the nearest 0.1 cm, using a flexible but inextensible measuring tape by using the perimeter of the point of greatest circumference around the hips and the WC as described above. The WHO classification for WHR was used in analysis (Normal: WHR<0.9 for male or WHR<0.8 for females, Android Obesity: WHR ≥ 0.9 for males or WHR ≥ 0.8 for females).

**Study procedure of data collection:** confidentiality, anonymity and privacy of all information were guaranteed at all the levels of this study. The survey was conducted in three faculties of state-owned universities offering general medicine as an option. After obtaining an ethical clearance from the National Bioethics Committee, administrative authorization was sought from the deans of the different faculties. A questionnaire developed for this study was used to collect data. The questionnaire was made of a section to collect data on socio-demographic factors and eating practices (dietary habits). Another was a “24 Hour Food Recall” to collect data and a last section to collect anthropometric data. Students were contacted with the help of class delegates. The theme of the study was introduced, and the objectives explained. Students who were willing to participate and who signed a consent form were given the questionnaire to complete. Anthropometric parameters were then measured (following procedures described above) in strict respect of personal privacy and recorded.

**Data analysis:** data was entered using Census and Survey Processing System (CSPro) version 5.0 and analyzed using Statistical Package for Social Sciences (SPSS) software version 18.0. Frequencies and percentages were determined for categorical variables. Means and standard deviations (mean ± SD) were calculated for continuous variables. To investigate associations between socio-demographic characteristics, dietary habits and the outcome variable (BMI) we used Chi-Square test as they were categorical variables. Fischer´s exact test was used in cases were conditions for Chi-Square test were not met. Statistical significance was set at p ≤ 0.05.

**Quality control measures:** some measures employed to improve on data quality included: training on how to take anthropometric measurements. The research questionnaires were pre-tested with 20 sixth year medical students of FMBS-University of Yaoundé I to assess difficulties in comprehension and time to complete the questionnaire. This permitted necessary ameliorations which essentially concerned use of appropriate vocabulary and improved validity of questions. Measuring equipment used in the study were checked regular and calibration done after each use. Measurements for each student repeated by the same researcher and the value used in analysis was the average value of the different readings.

## Results

**General characteristics of the participants:** a total of 203 students from the three state universities participated in the study with 52.7% from FMBS-UYI (n =107), 24.6% from FHS-UBa (n=50) and 22.7% from FHS-UB (n=46). Of these, 44.3% were males (n=90). The ages of the students ranged from 17 to 27 years with a mean age of 20.8 ± 1.7 years. Most (36.0%, n = 73) had monthly income (allowance) less than 20000FCFA (1USD = 580Frs) and majority (59.1%, n=128) lived alone (Table 1).

**Table 1:** Socio-demographic information of university students

Socio-demographic characteristics		FMSB-UYI		FHS-UBa		FHS-UB		Total	
		n=107	%	n=50	%	n=46	%	N=203	%
Gender	Male	53	50%	16	32%	21	46%	90	44%
	Female	54	50%	34	68%	25	54%	113	56%
	Total	107	100%	50	100%	46	100%	203	100%
Religion	Christian	92	86%	49	98%	42	91%	183	90%
	Islam	15	14%	1	2%	4	9%	20	10%
	Total	107	100%	50	100%	46	100%	203	100%
Monthly Allowance*	Less than 20000frs	35	33%	21	42%	17	37%	73	36%
	20000frs to less than 30000frs	24	22%	17	34%	17	37%	58	29%
	30000frs to less than 40000frs	20	19%	6	12%	7	15%	33	16%
	40000frs to less than 50000frs	11	10%	4	8%	3	7%	18	9%
	50000frs and more	17	16%	2	4%	2	4%	21	10%
	Total	107	100%	50	100%	46	100%	203	100%
Place of residence	University hostel	55	51%	33	66%	32	70%	120	59%
	With parents	52	49%	17	34%	14	30%	83	41%
	Total	107	100%	50	100%	46	100%	203	100%

* 1USD = 550frs

**Anthropometric measurements:** the mean weight of students was 65.09 ± 9.45Kg. Means for body mass index (BMI) and waist circumference (WC) were 23.13 ± 3.12Kg/m2, 77.46 ± 8.12cm respectively. In this study, the prevalence of underweight, overweight and obesity was 4.9%, 21.7% and 3.0% respectively (Table 2).

**Table 2:** Anthropometric profile of male and female students

Anthropometric variable		FMSB-UYI			FMSB-UYI			FMSB-UYI			Summary		
		Male (n=53)	Female (n=54)	Total (n=107)	Male (n=16)	Female (n=34)	Total (n=50)	Male (n=21)	Female (n=25)	Total (n=46)	Male (n=90)	Female (n=113)	Total (n=203)
**Body Mass Index (BMI)**	Underweight: BMI<18.5	4	4	8	1	0	1	0	1	1	5	5	10
	%	7.5%	7.4%	7.5%	6.3%	0.0%	2.0%	0.0%	4.0%	2.2%	5.6%	3.5%	4.9%
	Ideal: 18.5≤BMI<25.0	42	31	73	12	21	33	18	19	37	72	71	143
	%	79.2%	57.4%	68.2%	75.0%	61.8%	66.0%	85.7%	76.0%	80.4%	80.0%	3.5%	70.4%
	Overweight: 25.0≤BMI<30.0	6	17	23	3	11	14	3	4	7	12	32	44
	%	11.3%	31.5%	21.5%	18.8%	32.4%	28.0%	14.3%	16.0%	15.2%	13.3%	3.5%	21.7%
	Obese: BMI≥30	1	2	3	0	2	2	0	1	1	1	5	6
	%	1.9%	3.7%	2.8%	0.0%	5.9%	4.0%	0.0%	4.0%	2.2%	1.1%	3.5%	3.0%
	BMI ± SD (Kg/m2)	22.5	23.4	22.9	23.1	24.1	23.8	22.9	22.9	22.9	22.8	23.5	23.1
	SD	2.7	3.8	3.3	2.6	3.2	3.0	2.5	3.2	2.9	2.6	3.4	3.1
**Waist Circumference (cm)**	Normal: WC<94 for male or WC<80 for females	52	30	82	15	22	37	21	20	41	88	72	160
	%	98.1%	55.6%	76.6%	93.8%	64.7%	74.0%	100.0%	80.0%	89.1%	97.8%	3.5%	78.8%
	Android Obesity: WC ≥94 for males or WC ≥80 for females	1	24	25	0	18	18	1	14	15	2	56	58
	%	1.9%	44.4%	23.4%	0.0%	52.9%	36.0%	4.8%	56.0%	32.6%	2.2%	3.5%	28.6%
	WC (cm)	77.1	78.7	77.9	76.3	78.0	77.5	76.8	76.2	76.5	76.7	77.6	77.5
	SD	7.4	9.5	8.5	7.5	10.0	9.2	4.1	6.8	5.7	6.3	8.8	8.1
**Waist Hip Ratio**	Normal: WHR<0.9 for male or WHR<0.8 for females	52	28	80	16	16	32	20	11	31	88	55	143
	%	98.1%	51.9%	74.8%	100.0%	47.1%	64.0%	95.2%	44.0%	67.4%	97.8%	3.5%	70.4%
	Android Obesity: WHR ≥0.9 for males or WHR ≥0.8 for females	1	24	25	0	18	18	1	14	15	2	56	58
	%	1.9%	44.4%	23.4%	0.0%	52.9%	36.0%	4.8%	56.0%	32.6%	2.2%	3.5%	28.6%
	WHR	0.82	0.79	0.81	0.81	0.80	0.80	0.81	0.78	0.80	0.81	0.79	0.80
	SD	0.04	0.05	0.05	0.03	0.05	0.04	0.04	0.04	0.04	0.04	0.05	0.04

**Figure 1:**
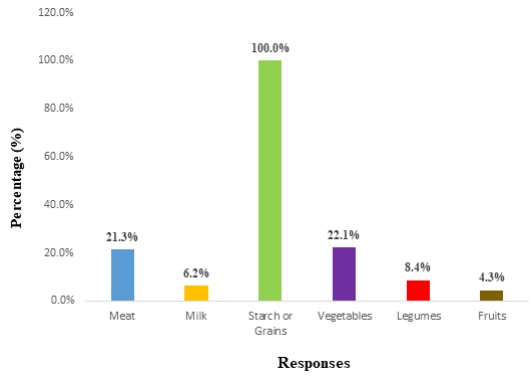
Food group consumption of students

**Dietary habits (eating practices):** from the 24 Hour Food Recall, 16.7% (n=34) students had one meal, 49.8% (n=101) had two meals, 33.5% (n=68) had three meals. In addition, 40.8% (n=83) had taken a snack (“snack” refers to all foods and drinks taken outside the context of the three main meals) in a day. Breakfast was consumed by 18.7% (n=38) of students in a day, while 29.6% (n=60) had lunch and 51.7% (n=105) had supper. Grouping reported food items consumed according to major food groups, 21.3% of the students ate meat daily, 6.2% took milk or dairy products, 100.0% of the students ate starch daily, 4.3% ate a fruit daily (Figure 1). Concerning meal consumption tendencies, 33.0% (n=67) of the students regularly took breakfast, 48.2% (n=98) regularly took lunch and 60.6% (n=123). The most frequently reported (52.7%, n=214) reason for skipping a meal being by students being attributed to being busy. Of these students, 63.0% (n=128) admitted consuming alcohol either occasionally or regularly. Fruits were regularly eaten by 26.1% (n=53) of the students and 25.1% (n=51) seldom or never ate fruits. Meanwhile, vegetable was regularly consumed by 24.6% (n=50) (Table 3).

**Table 3:** Frequency of different eating practices by BMI Class

Characteristics		Underweight (n=10)	Ideal(n=143)	Overweight (n=44)	Obese(n=6)	P-Value
Gender	Male	5	72	12	1	0.023*
	%	50	50.3	27.3	16.7	
	Female	5	71	32	5	
	%	50	49.7	72.7	83.3	
Breakfast consumption	Regularly	4	49	13	1	0.798
	%	40	34.3	29.5	16.7	
	Sometimes	2	60	19	3	
	%	20	42	43.2	50	
	Seldom	4	32	11	2	
	%	40	22.4	25	33.3	
	Never	0	2	1	0	
	%	0	1.4	2.3	0	
Lunch consumption	Regularly	5	69	22	2	0.819
	%	50	48.3	50	33.3	
	Sometimes	5	55	15	4	
	%	50	38.5	34.1	66.7	
	Seldom	0	16	7	0	
	%	0	11.2	15.9	0	
	Never	0	3	0	0	
	%	0	2.1	0	0	
Supper consumption	Regularly	5	90	24	4	0.289
	%	50	62.9	54.5	66.7	
	Sometimes	3	39	16	1	
	%	30	27.3	36.4	16.7	
	Seldom	1	14	4	0	
	%	10	9.8	9.1	0	
	Never	1	0	0	0	
	%	10	0	0	0	
Fruits consumption	Regularly	3	36	12	2	0.97
	%	30	25.2	27.3	33.3	
	Sometimes	6	70	20	3	
	%	60	49	45.5	50	
	Seldom	1	34	12	1	
	%	10	23.8	27.3	16.7	
	Never	0	3	0	0	
	%	0	2.1	0	0	
Vegetables consumption	Regularly	2	34	11	3	0.688
	%	20	23.8	25	50	
	Sometimes	7	81	22	3	
	%	70	56.6	50	50	
	Seldom	1	28	11	3	
	%	10	19.6	25	50	
	Never	0	0	0	0	
	%	0	0	0	0	
Snack consumption	Consume snacks	9	115	40	0	0.234
	%	90	80.4	90.9	0	
	Does not take snacks	1	28	4	6	
	%	10	19.6	9.1	100	
Alcohol consumption	Consume alcohol	4	89	33	0	0.057
	%	40	62.2	75	0	
	Does not take alcohol	6	54	11	33.3	
	%	60	37.8	25	66.7	

**Association between eating habits, sociodemographic factors and body mass index categories (BMI):** gender of student was statistically significantly associated with BMI with more females than males being either overweight or obese. Category and gender showed significantly more females were overweight or obese compared to males in the study (Table 3).

## Discussion

The results of our study showed that meal skipping was common among the students as just 33.5% had three meals a day from the 24-hour food recall and most (49.8%) had two meals. Supper was the most consumed meal (78.8%) while breakfast was the least consumed (18.7%). This was corroborated with observation that supper and breakfast were most and least respectively regularly consumed by students. Frequency of consumption of fruits, milk and dairy product, vegetable, legumes and meat by students was poor. These findings indicate it is likely that medical students do not eat balanced meals and do not meet their daily nutrient requirements for it is recommended to eat a fruit daily and vegetables regularly in addition to eating three main meals a day in order to meet the daily nutrients and energy requirements [5]. In this study, snacking was common as 40.8% of students reported eating a snack on the 24-Hour Food Recall. Alcohol (ethanol) consumption was a common trait among medical students for 63.0% reported consuming alcohol. Our findings are similar to that of studies in Malaysia [8] where most students had two meals a day and breakfast consumption among students was poor with 58.5% of skipping it.

Similarly, snacking was a daily habit in about one third of Saudi students [9] which is similar to the 40.8% in our study. In contrast to our findings, snacking was not a common practice among Chinese college student. Only about one tenth of Chinese college students take snacks daily [10]. Poor intake of vegetables and fruits a finding in our study was also common among Saudi students as just 32.2% and 36.1% ate vegetables or a fruit weekly [9]. Our findings are consistent with the results of a study of university students in Douala, Cameroon [3] that also showed university students ate very little fruits and vegetables. Contrary to African medical school students, 83.5% of Asian (Chinese) college students consumed fruits and vegetables daily [10]. This could probably be explained by the vegetarian tradition of Asian cuisine. Similar trends in eating practices characterized by low vegetable, fruit and milk consumption, meal skipping had been described among medical students in Croatia [11] and university students in Florida [12]. These poor habits of meal skipping, frequent alcohol consumption, low fruits, meat, vegetables and legumes consumption are to be discouraged amongst these students since these practices may have a negative effect on their health in later years.

The nutritional status of the students was assessed by anthropometric measurement of BMI, WC, WHR. Anthropometric assessments: BMI, WC, WHR includes an appraisal of the patient´s weight, height and body composition, that is, lean body mass, fat stores and distribution [13]. The prevalence of underweight, overweight and obesity in our study was 4.9%, 21.7% and 3.0% respectively. This implied using BMI as a measurement of nutritional status, 29.6% of the students were malnourished. This trend was consistent with that of a similar study in Egypt were 41.3%, 9.5%, 36.9%, 12.5% of medical students were of normal weight, underweight, overweight and obese respectively [14] and in Cameroon were 21.8% and 15.7% of university masters level students were overweight and obese respectively [3]. However, underweight tends to be higher compared to overweight or obesity in Asians [8,15]. The variations in these study and others reflect differences in the severity of obesity problems among young adults across nations. Using waist circumference (WC) and waist-hip ratio (WHR), the prevalence of android obesity was 21.2% and 29.5% respectively using the WHO classification [16].

The WHR appears to overestimate the prevalence of android obesity as many people with normal BMI were included when it is used. Gender was associated with BMI (p=0.026). More female students were significantly either overweight or obese compared to male counterparts. This could be explained from our data by the observation that a significant number of females (n=41) than males (n=2), using waist circumference, had abdominal obesity. This was similar to findings in Cameroon [10] and other similar studies [8,17]. There was not a significant association between number of meals, monthly allowance, living condition, snacking, fruits, vegetable and alcohol consumption with BMI in our study.

## Conclusion

In this study, second year medical students in state universities were shown to have poor eating habits characterized by meal skipping, snacking, low fruit, vegetable and milk consumption and many of them did consume alcohol. The prevalence of malnutrition using BMI as the index was 29.5% with more females affected more than males. This study which is the first of its kind among medical school students in Cameroon was with some limitations. These included possibilities of recall bias inherent to the nature of a 24-Hour Food Recall questionnaire. Based on our findings, we recommend the need for efforts to promote healthy dietary practices among youths, particularly among females, as a combat malnutrition. Though not a direct objective of this study, educating and empowering medical student on healthy eating and living may be beneficial as they have the potential of helping others make healthy dietary and lifestyle choices as future medical personnel and hence they have a role to play in combating malnutrition.

### What is known about this topic

- Prevalence of overweight and obesity among University of Buea students, Cameroon;
- Relationship of physical health status and health practices;
- Analysis of eating habits according to socio-demographic characteristics of college students.

### What this study adds

- There are poor eating habits characterized by meal skipping, snacking, low fruit, vegetable and milk consumption and many of them did consume alcohol;
- The prevalence of malnutrition using BMI as the index was 29.5% with more females affected more than males. BMI was significantly associated with type of meal (supper).
